# The Unfortunate Poor

**Published:** 1869-03

**Authors:** 


### THE UNFORTUNATE POOR.

A GENTLEMAN, in dress, language and appearance, came to me recently, on a Saturday evening, and said neither himself nor wife had eaten anything that day, and wanted the loan of a small amount of money until the next Monday morning; we were greatly surprised. Hundreds every night are glad to sleep on hard wooden benches in our warm prison-stations; what multitudes of mothers and children go to bed hungry every night, in this city alone; who can tell! But a still larger multitude impose upon the generosity of our natures, who are utterly unworthy of sympathy or aid; these are among the street beggars and those who habitually besiege our kitchen doors, from dawn till dark, and many times into the late hours of the night; they throng our business offices, and their importunity is often painful. The rule as to our dwellings ought to be rigidly enforced—never allow anything to be given, received, bought or sold by our servants at our doors; tell them they may give of their own money as largely and as often as they please, but never give what our money pays for without our consent, for it is theft. Let every reader decide what amount he wants to give to the poor, take it to the Bible House, Room 39, third floor, entrance on Eighth street; tickets will be furnished him on which will be found the residence and name of a responsible person near his dwelling; when applied to for charity, write your name on the ticket and give it to the applicant to hand to the address indicated; this person will go with the applicant to his place of abode, will minutely inquire into his circumstances and character and habits, and if found really deserving adequate aid is judiciously afforded. Those who are really worthy and are really in need, will deliver the tickets, for they have nothing to fear from investigation and are sure of reasonable help; the unworthy will never deliver the tickets, and when they find that they never get anything but tickets at your house, they very soon cease to come; and a happy riddance it is, because they no longer call your servants from their work, nor tempt them to steal your provisions to supply the wants of the unprincipled, the lazy and the improvident.

Indiscriminate giving is a great demoralizer; it encourages laziness, lying and improvidence, and injures both giver and receiver. It does seem that no better method can be devised to help the worthy poor, and many of the best names in the city are interested in the management.

